# Relationship between spectrotemporal modulation detection and music perception in normal-hearing, hearing-impaired, and cochlear implant listeners

**DOI:** 10.1038/s41598-017-17350-w

**Published:** 2018-01-15

**Authors:** Ji Eun Choi, Jong Ho Won, Cheol Hee Kim, Yang-Sun Cho, Sung Hwa Hong, Il Joon Moon

**Affiliations:** 1 Department of Otorhinolaryngology-Head and Neck Surgery, Dankook University Hospital, Cheoan, 31116 Republic of Korea; 2 Division of Ophthalmic and Ear, Nose and Throat Devices, Office of Device Evaluation, Center for Devices and Radiological Health, US Food and Drug Administration, Silver Spring, Maryland 20993 USA; 3 Hearing Research Laboratory, Samsung Medical Center, Seoul, 06351 Republic of Korea; 4 Department of Otorhinolaryngology-Head and Neck Surgery, Samsung Medical Center, Sungkyunkwan University School of Medicine, Seoul, 06351 Republic of Korea; 5 Department of Otorhinolaryngology-Head and Neck Surgery, Samsung Changwon Hospital, Sungkyunkwan University School of Medicine, Changwon, 51353 Republic of Korea

**Keywords:** Inner ear, Transduction

## Abstract

The objective of this study was to examine the relationship between spectrotemporal modulation (STM) sensitivity and the ability to perceive music. Ten normal-hearing (NH) listeners, ten hearing aid (HA) users with moderate hearing loss, and ten cochlear Implant (CI) users participated in this study. Three different types of psychoacoustic tests including spectral modulation detection (SMD), temporal modulation detection (TMD), and STM were administered. Performances on these psychoacoustic tests were compared to music perception abilities. In addition, psychoacoustic mechanisms involved in the improvement of music perception through HA were evaluated. Music perception abilities in unaided and aided conditions were measured for HA users. After that, HA benefit for music perception was correlated with aided psychoacoustic performance. STM detection study showed that a combination of spectral and temporal modulation cues were more strongly correlated with music perception abilities than spectral or temporal modulation cues measured separately. No correlation was found between music perception performance and SMD threshold or TMD threshold in each group. Also, HA benefits for melody and timbre identification were significantly correlated with a combination of spectral and temporal envelope cues though HA.

## Introduction

Speech understanding in hearing impaired listeners fitted with hearing devices has been gradually improved due to advancement in technology of hearing aid (HA) or cochlear implant (CI)^1,2^. Despite such advancement, the majority of people wearing HAs or CI complain reduced quality of music they hear through their devices^3,4^. Thus, HA or CI users often express the need to optimize their hearing devices for better music perception qualities^5,6^.

Fundamental elements of music perception have been generally accepted as perceiving pitch, melody, timbre, and rhythm in music. A series of studies have shown that not only music amusement, but also perceiving certain elements of music remain challenging for many HA or CI users^3,4,7^. Looi *et al*. (2008)^7^ have compared these four key elements of music perception in 15 CI users, 15 HA users, and 10 normal hearing (NH) listeners and found that HA and CI users could perceive musical rhythm similar to NH listeners. However, HA and CI users showed worse performance than NH listeners in the perception tests of pitch, melody, and timbre^7^. This might be due to the fact that prescription rules of HAs or CI coding strategies are primarily designed for speech, particularly in quiet listening environments, not for music listening^8–10^.

In order to develop technology for hearing devices to have better music perception outcomes, it is important to understand how specific acoustic elements contribute to music perception. Previous studies have evaluated the contribution of spectral and temporal sensitivity to music perception performance in CI users^10–13^. For example, Won *et al*. (2010)^11^ have reported that better spectral resolution measured by spectral-ripple discrimination contributes to better music perception in CI users. Kong *et al*. (2004)^13^ have demonstrated that both temporal and spectral cues contribute to melody recognition while CI users have mostly relied on the rhythmic cues for melody recognition. However, previous studies measured spectral or temporal modulation sensitivities separately. To the best of our knowledge, no study has reported the ability of using combined spectral and temporal modulation cues in the same stimulus to examine their potential relationship with music perception abilities in hearing impaired listeners using HAs or CIs. A combination of spectral and temporal modulation cues, often called “spectrotemporal modulation (STM)” cues, represent spectral patterns that change over time or temporal modulation patterns that differ across frequency channels^14–16^. Because dynamic spectral and temporal information is necessary to fully describe music, we hypothesized that a combination of spectral and temporal modulation cues would be correlated with music perception more compared to spectral or temporal modulation cues separately. Thus, the primary goal of the present study was to measure music perception abilities using three different psychoacoustic tests including spectral modulation detection (SMD) test, temporal modulation detection (TMD) test, and STM detection test in NH listeners, HA users and CI users with their own devices and examine the relationship between psychoacoustic and music perception abilities. Music perceptions were compared between unaided and aided conditions of HA users. In addition, psychoacoustic mechanisms related to HA benefit for music perception (the difference of music perception abilities between aided and unaided conditions) was investigated.

## Results

### Psychoacoustic performance for NH listeners, HA users, and CI users

**Figure 1:**
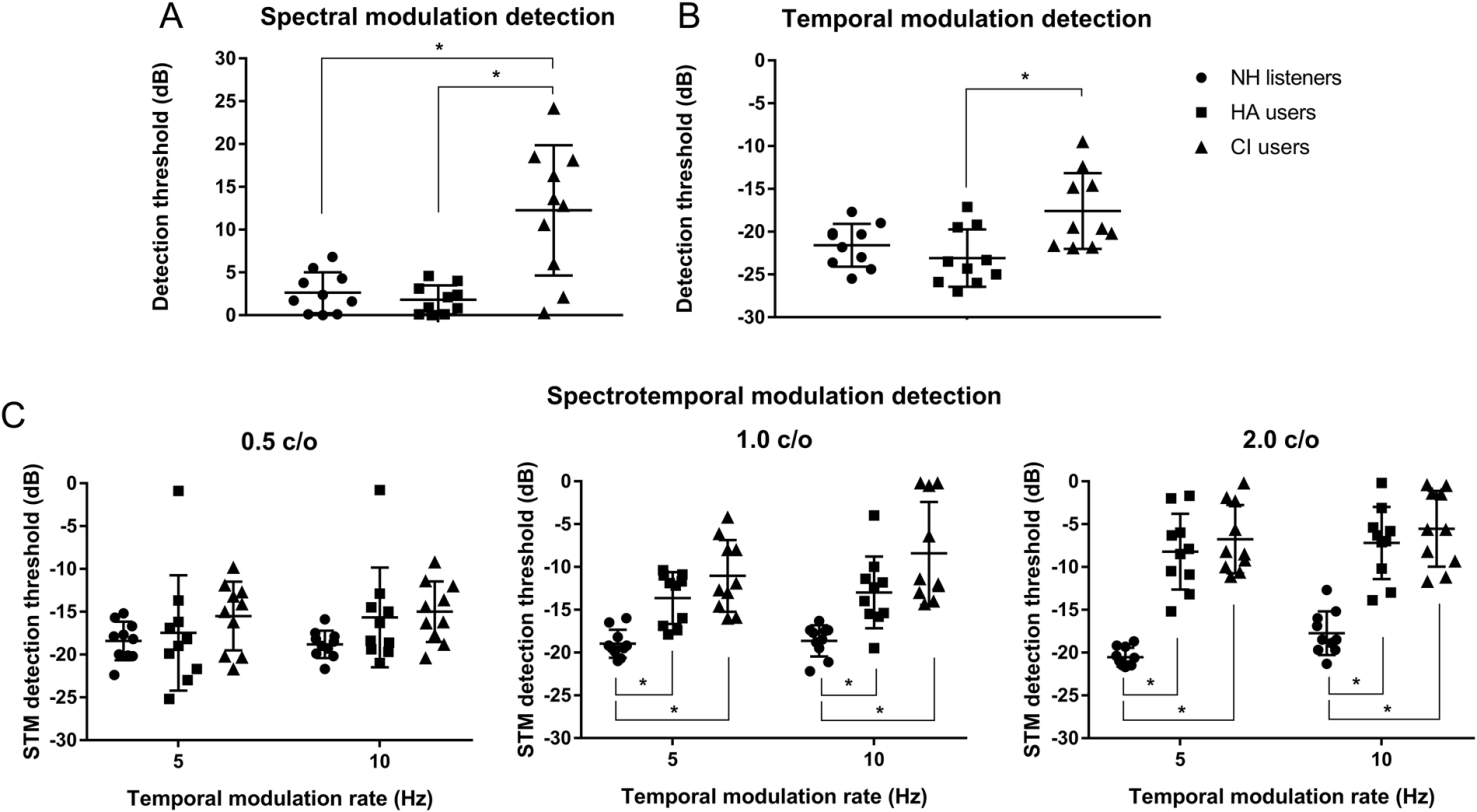
Psychoacoutic performances of each subject. Results of detection thresholds (dB) for each stimuli condition for NH listeners, HA users, and CI users are shown in circle (•), square (■), and triangle (▲), respectively. Spectral modulation detection (SMD) threshold (**A**) and temporal modulation detection (TMD) threshold (**B**) are shown in the upper row. Spectrotemoral modulation (STM) detection thresholds (**C**) are shown in the lower row. STM detection thresholds for spectral rates of 0.5, 1.0, and 2.0 c/o are shown in the left, middle, and right columns, respectively. Bars and error bars represent mean detection thresholds and standard deviation. Asterisk (*) indicates significant difference between two groups in post-hoc analysis (adjusted P-value was 0.05/3 based on Bonferroni correction).

Scatter plots of psychoacoustic performance for NH listeners, HA users, and CI users are shown in Fig. 1. SMD thresholds for a spectral density of 1 c/o are shown in Fig. 1A. Here, lower detection thresholds indicate better SMD performance. NH listeners and HA users showed similar performance on SMD test (p = 0.920). However, CI users performed significantly worse than both NH listeners and HA users on the SMD test (both p < 0.001).

TMD thresholds at 10 Hz are shown in Fig. 1B. For the TMD test, more negative detection thresholds imply better TMD performance. Results of TMD test showed similar patterns to those observed in SMD test. NH listeners showed similar performance on TMD test compared to HA users (p = 0.615) and CI users (p = 0.043). However, CI users performed significantly (p = 0.005) worse than HA users on TMD test.

STM detection thresholds for six different stimuli conditions across three subjects groups are shown in Fig. 1C. More negative STM thresholds indicate better STM detection performance. Overall, there were differences in STM detection thresholds among the three subject groups, indicating that different hearing mechanisms could affect STM detection performance. One way ANOVA results showed that there was a significant effect of subject group on STM detection thresholds at spectral densities of 1.0 c/o [F(2,27) = 16.83, p < 0.001 for 5 Hz; F(2,27) = 13.73, p < 0.001 for 10 Hz] and 2.0 c/o [F(2,27) = 46.71, p < 0.001 for 5 Hz; F(2,27) = 29.88, p < 0.001 for 10 Hz], but not at lower spectral density of 0.5 c/o [F(2,27) = 0.976, p = 390 for 5 Hz; F(2,27) = 2.547, p = 0.097]. Post-hoc analysis showed that STM detection thresholds for NH subjects were significantly lower (i.e., better performance) than both HA users and CI users at spectral densities of 1.0 and 2.0 c/o. Between HA users and CI users, there was no significant difference in performance at any STM stimulus condition.

### Music perception performances for NH listeners, HA users, and CI users

**Figure 2:**
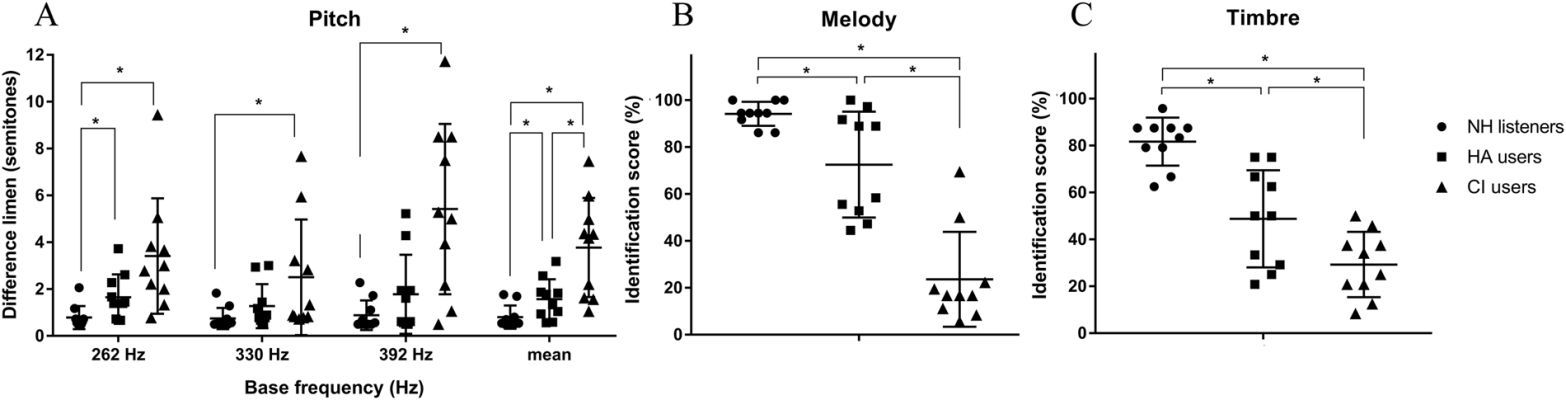
Music perception abilities for each subject. Results of music perception abilities for NH listeners, HA users, and CI users are shown in circle (•), square (■), and triangle (▲), respectively. Bars and error bars represent mean abilities and standard deviation. Asterisk (*) indicates significant difference between two groups in post-hoc analysis (adjusted P-value was 0.05/3 based on Bonferroni correction).

Scatter plots of music perception for the three subject groups are shown in Fig. 2. The mean pitch-direction discrimination score was 0.8 ± 0.5 semitones for NH listeners, 1.6 ± 0.8 semitones for HA users, and 3.8 ± 2.1 semitones for CI users (Fig. 2A). Kruskal-Wallis test results showed a significant effect of subject group on pitch-direction discrimination ability [H(2) = 15.749, p < 0.001]. Post-hoc analysis confirmed that there were significant differences in mean pitch-direction discrimination scores between two different subject groups (i.e., NH listeners vs. HA users, HA users vs. CI users, and CI users vs. NH listeners).

The mean melody identification score was 94.2 ± 5.1% for NH listeners, 72.5 ± 22.5% for HA users, and 23.6 ± 20.2% for CI users (Fig. 2B). One-way ANOVA showed that there was a significant effect of subject group on melody identification ability [F(2,27) = 41.542, p < 0.001]. Post-hoc analysis confirmed that there were significant differences in melody identification scores between two different subject groups (i.e., NH listeners vs. HA users, HA users vs. CI users, and CI users vs. NH listeners).

Results of timbre identification test showed similar patterns to those of melody identification test. NH subjects showed a mean score of 81.7 ± 10.2%. HA and CI subjects showed a mean score of 48.8 ± 13.9% and 29.2 ± 13.9%, respectively. One-way ANOVA showed that there was a significant effect of subject group on timbre identification ability [F(2,27) = 28.959, p < 0.001]. Timbre identification abilities were also significantly different between two different subject groups (i.e., NH listeners vs. HA users, HA users vs. CI users, and CI users vs. NH listeners).

### Correlations between psychoacoustic performances and music perceptions for all participants

**Figure 3:**
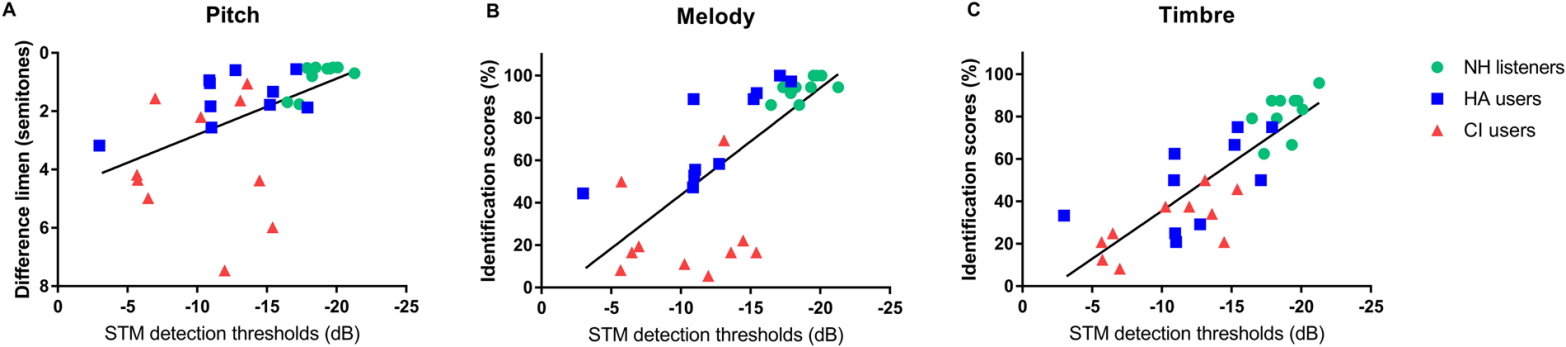
Scatter plots of mean spectrotemporal modulation (STM) detection thresholds and music abilities. X-axis represents music perception abilities. Y-axis represents mean STM detection test. Mean STM detection thresholds defines averaged thresholds across six different stimuli conditions. Results of scatter plots for NH listeners, HA users, and CI users are shown in green circle (•), blue squared (■), and red triangle (▲), respectively. Panel A indicates pitch discrimination scores. Panel B indicates melody identification scores. Panel C indicates timbre identification sores.

Correlations of SMD, TMD, and STM detection thresholds with all music perception performances for all participants are shown in Table 1. Psychoacoustic performances were significantly correlated with music perception performances except for that between TMD thresholds and timbre identification scores. STM detection thresholds showed higher correlations with all music perception scores than SMD or TMD thresholds. Scatter plots of mean STM detection thresholds and music perception abilities in all three subject groups are shown in Fig. 3.

**Table 1:** The Pearson correlation coefficients and significant values between psychoacoustic and music perception performances.

Variables	Music perceptions					
	Pitch		Melody		Timbre	
	*R*	*p*	*R*	*p*	*R*	*p*
**SMD**	**0.376**	**0.040**	**−0.507**	**0.004**	**−0.532**	**0.002**
**TMD**	**0.450**	**0.013**	**−0.369**	**0.045**	−0.329	0.076
**STM detection (mean)**	**0.520**	**0.003**	**−0.720**	**<0.001**	**−0.837**	**<0.001**
**0.5 c/o, 5 Hz**	**0.426**	**0.019**	**−0.427**	**0.019**	**−0.467**	**0.009**
**0.5 c/o, 10 Hz**	**0.463**	**0.010**	**−0.630**	**<0.001**	**−0.592**	**0.001**
**1.0 c/o, 5 Hz**	**0.469**	**0.009**	**−0.721**	**<0.001**	**−0.844**	**<0.001**
**1.0 c/o, 10 Hz**	**0.669**	**<0.001**	**−0.809**	**<0.001**	**−0.900**	**<0.001**
**2.0 c/o, 5 Hz**	**0.626**	**<0.001**	**−0.757**	**<0.001**	**−0.822**	**<0.001**
**2.0 c/o, 10 Hz**	**0.430**	**0.018**	**−0.688**	**<0.001**	**−0.824**	**<0.001**

The bold indicates significant difference at the level of 0.05.

### Relationships among psychoacoustic performances and music perceptions in each subject group

Simple linear regression analyses were performed to investigate the relationship between psychoacoustic performances and music perception scores in each subject group. For NH subjects, all music perception abilities were significantly correlated with STM detection performance (Table 2). Pitch discrimination and timbre identification scores were significantly correlated with the low spectral modulation condition (0.5 c/o) of the STM detection test while melody identification scores were significantly correlated with the high spectral modulation condition (2.0 c/o) of the STM detection test.

**Table 2:** Results of simple linear regression analyses between psychoacoustic performances and music perceptions abilities for each subgroup.

Variable	Pitch				Melody				Timbre			
	Slope	95% CI	*p*-value	R^2^	Slope	95% CI	*p*-value	R^2^	Slope	95% CI	*p*-value	R^2^
**NH subjects**												
**SMD**	0.123	−0.012, 0.259	0.069	0.356	−1.071	−2.594, 0.452	0.144	0.247	−0.784	−4.219, 2.650	0.613	0.034
**TMD**	0.114	−0.017, 0.245	0.081	0.333	−1.083	−2.503, 0.336	0.116	0.279	−2.053	−4.928, 0.822	0.138	0.253
**STM detection (mean)**	**0.238**	**0.033, 0.443**	**0.028**	**0.473**	−2.24	−4.543, 0.063	0.055	0.386	−3.668	−8.694, 1.359	0.131	0.261
**0.5 c/o, 5 Hz**	0.129	−0.012, 0.270	0.068	0.358	−0.099	−1.930, 1.732	0.904	0.002	−2.355	−5.455, 0.745	0.118	0.277
**0.5 c/o, 10 Hz**	**0.211**	**0.029, 0.394**	**0.029**	**0.471**	−1.175	−3.607, 1.257	0.298	0.134	**−5.053**	**−8.228, −1.877**	**0.006**	**0.627**
**1.0 c/o, 5 Hz**	0.144	−0.073, 0.360	0.165	0.226	−1.911	−3.945, 0.123	0.062	0.37	−1.259	−6.254, 3.736	0.577	0.041
**1.0 c/o, 10 Hz**	0.078	−0.131, 0.286	0.415	0.085	−1.439	−3.380, 0.501	0.126	0.268	−2.992	−6.788, 0.805	0.107	0.292
**2.0 c/o, 5 Hz**	0.223	−0.087, 0.533	0.136	0.256	**−3.672**	**−5.918, −1.426**	**0.006**	**0.64**	−1.134	−8.523, 6.255	0.733	0.015
**2.0 c/o, 10 Hz**	0.115	−0.011, 0.241	0.068	0.357	**−1.396**	**−2.572, −0.219**	**0.026**	**0.483**	−0.676	−3.886, 2.534	0.64	0.029
**HA users**												
**SMD**	−0.29	−0.624, 0.045	0.081	0.333	2.877	−7.745, 13.498	0.55	0.047	0.23	−9.754, 10.214	0.959	**<**0.001
**TMD**	−0.027	−0.232, 0.178	0.768	0.012	1.914	−3.338, 7.166	0.425	0.081	−0.112	−5.141, 4.918	0.961	**<**0.001
**STM detection (mean)**	0.113	−0.019, 0.245	0.085	0.326	**−4.17**	**−6.754, −1.586**	**0.006**	**0.634**	−2.815	−5.993, 0.364	0.075	0.343
**0.5 c/o, 5 Hz**	0.073	−0.011, 0.157	0.081	0.333	−1.99	−4.183, 0.203	0.07	0.354	−1.353	−3.601, 0.896	0.203	0.194
**0.5 c/o, 10 Hz**	0.09	−0.004, 0.183	0.058	0.38	−2.272	−4.826, 0.282	0.074	0.345	−1.259	−3.968, 1.449	0.315	0.126
**1.0 c/o, 5 Hz**	0.068	−0.153, 0.289	0.499	0.059	**−6.354**	**−9.491, −3.217**	**0.002**	**0.732**	**−4.957**	**−8.776, −1.138**	**0.017**	**0.528**
**1.0 c/o, 10 Hz**	0.117	−0.018, 0.252	0.081	0.333	**−4.056**	**−6.955, −1.157**	**0.012**	**0.565**	**−3.28**	**−6.305, −0.256**	**0.037**	**0.439**
**2.0 c/o, 5 Hz**	0.077	−0.066, 0.221	0.249	0.162	**−4.585**	**−6.408, −2.761**	**<0.001**	**0.808**	−2.576	−5.765, 0.613	0.1	0.303
**2.0 c/o, 10 Hz**	0.112	−0.025, 0.249	0.096	0.309	**−4.369**	**−6.908, −1.830**	**0.004**	**0.663**	**−3.142**	**−6.234, −0.049**	**0.047**	**0.407**
**CI users**												
**SMD**	−0.071	−0.289, 0.148	0.477	0.065	0.995	−1.008, 2.998	0.285	0.141	−0.8	−2.138, 0.538	0.205	0.192
**TMD**	0.104	−0.276, 0.485	0.545	0.048	0.381	−3.335, 4.097	0.819	0.007	−0.175	−2.739, 2.390	0.879	0.003
**STM detection (mean)**	−0.024	−0.472, 0.424	0.904	0.002	−0.184	−4.473, 4.104	0.924	0.001	**−2.548**	**−4.650, −0.446**	**0.023**	**0.494**
**0.5 c/o, 5 Hz**	0.173	−0.234, 0.579	0.356	0.107	−0.84	−4.899, 3.218	0.646	0.028	−2.063	−4.345, 0.219	0.071	0.352
**0.5 c/o, 10 Hz**	−0.13	−0.610, 0.350	0.551	0.046	−1.173	−5.780, 3.433	0.573	0.041	−2.441	−4.998, 0.115	0.059	0.377
**1.0 c/o, 5 Hz**	−0.054	−0.464, 0.357	0.771	0.011	−0.086	−4.035, 3.864	0.961	**<**0.001	**−2.322**	**−4.276, −0.368**	**0.026**	**0.484**
**1.0 c/o, 10 Hz**	0.043	−0.241, 0.327	0.736	0.015	−0.143	−2.877, 2.590	0.907	0.002	**−1.663**	**−2.972, −0.354**	**0.019**	**0.518**
**2.0 c/o, 5 Hz**	−0.008	−0.439, 0.423	0.967	**<**0.001	0.305	−3.811, 4.421	0.869	0.004	−1.929	−4.294, 0.4367	0.097	0.307
**2.0 c/o, 10 Hz**	−0.194	−0.549, 0.161	0.243	0.166	0.68	−2.993, 4.353	0.681	0.022	−1.571	−3.786, 0.644	0.141	0.251

The bold indicates significant difference at the level of 0.05.

For HA users, melody and timbre identification scores were significantly correlated with STM detection thresholds (Table 2). However, pitch identification scores were not correlated with STM detection thresholds. Melody identification score showed the strongest correlation with STM detection thresholds at 2.0 c/o and 5 Hz (R2 = 0.808, p < 0.001). Timbre identification score showed the strongest correlation with STM detection thresholds at 1.0 c/o and 5 Hz (R2 = 0.528, p = 0.017). For CI users, only timbre identification scores were significantly correlated with SMT detection thresholds (Table 2). Timbre identification scores showed the strongest correlation with STM detection thresholds at 1.0 c/o and 10 Hz (R2 = 0.518, p = 0.019).

### Hearing aid benefit for music perception and associated psychoacoustic factors

Music perception abilities were compared between unaided and aided conditions for HA users (Supplement 1). Significantly better pitch discrimination and melody identification were found at aided condition compared to those at unaided condition (p = 0.012 for pitch discrimination; p = 0.015 for melody identification). However, timbre identification did not show significant difference between unaided and aided conditions.

**Figure 4:**
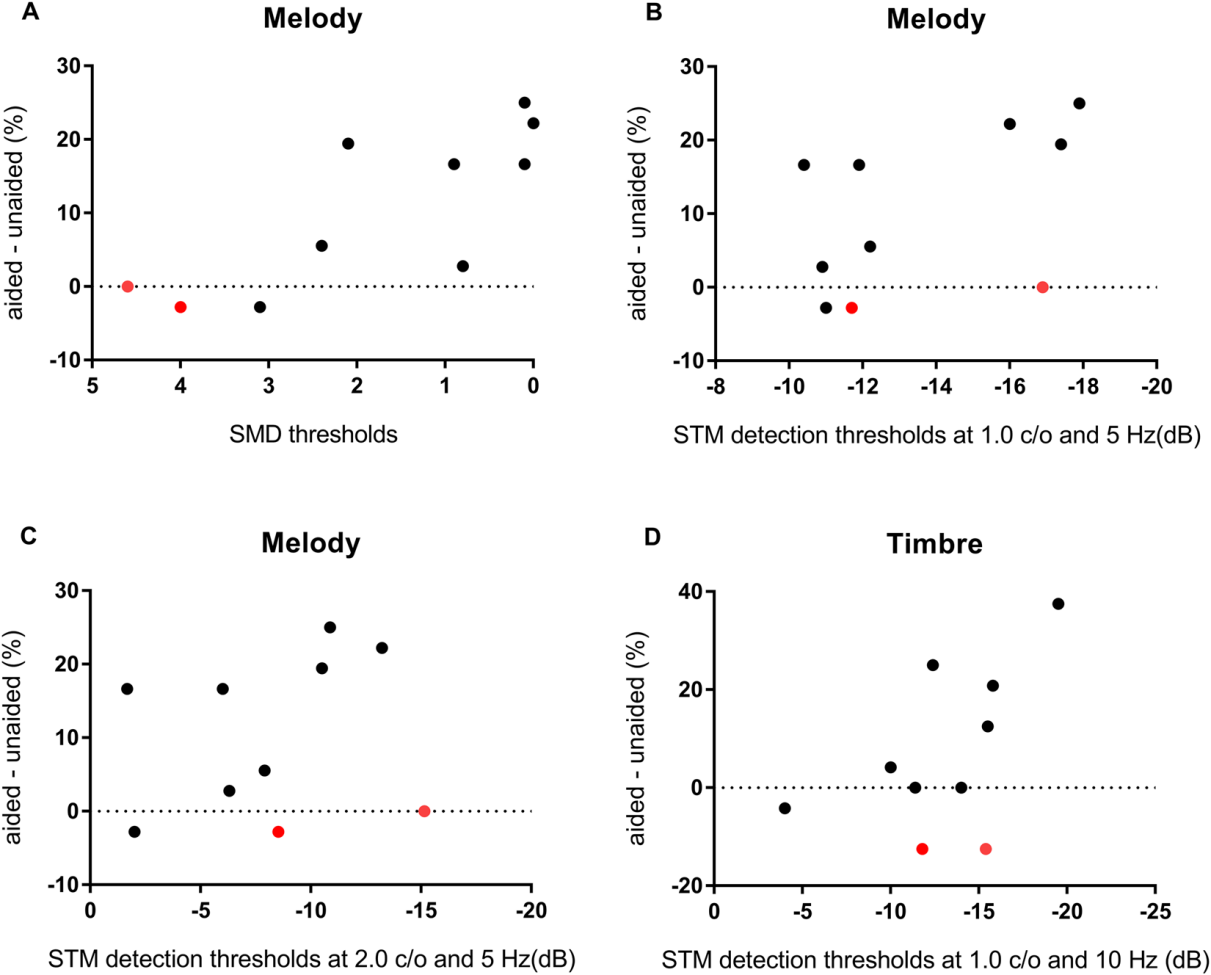
Scatter plots of difference in music perception performances between aided and unaided conditions and aided psychoacoustic performances. X-axis represents psychoacoustic thresholds. Y-axis represents difference in music perception between unaided and aided conditions. If music perception was improved after wearing HA, values of Y-axis are positive. Values of X-axis going to the right side means better psychoacoustic performance. Red circle indicates subjects with better unaided scores than limit of 95% confidence interval (1.35 semitones for pitch-direction discrimination, 78% for melody identification scores, and 55% for timbre identification scores).

To better understand psychoacoustic factors that might contribute to HA benefit for music perception ability, the relationship between aided psychoacoustic measures and differences in music perception abilities was evaluated at unaided and aided conditions. Since good performers at unaided condition might show less improvement in music perception after wearing a HA due to ceiling effect, partial correlation analysis was performed to control for unaided music perception scores. Results of partial correlation coefficients of aided psychoacoustic measures and HA benefit for music perception (the difference of music perception abilities between aided and unaided conditions) after controlling for unaided music perception scores are shown in Table 3. Scatter plots of aided psychoacoustic performances and HA benefit for music perception are shown in Fig. 4 with red circle indicating subjects who had better unaided scores than limit of 95% confidence interval (1.36 semitones for pitch-direction discrimination, 78% for melody identification scores and 55% for timbre identification scores). Figure 4 presents only significantly correlated results between psychoacoustic performances and HA benefit for music perception in partial correlation analysis.

**Table 3:** Partial correlations between changes of music perception performances and spectrotemporal modulation (STM) performances after controlling music perception abilities without a HA.

Variable	Pitch changes		Melody changes		Timbre changes	
	*R*	*p*	*R*	*p*	*R*	*p*
**SMD**	−0.326	0.392	**0.835**	**0.005**	0.415	0.267
**TMD**	0.142	0.715	0.567	0.112	0.245	0.525
**STM detection (mean)**	−0.533	0.139	−0.552	0.124	−0.542	0.132
**0.5 c/o, 5 Hz**	−0.470	0.201	−0.334	0.380	−0.523	0.149
**0.5 c/o, 10 Hz**	−0.419	0.261	−0.313	0.413	−0.409	0.274
**1.0 c/o, 5 Hz**	−0.468	0.204	**−0.848**	**0.004**	−0.639	0.064
**1.0 c/o, 10 Hz**	−0.655	0.056	−0.597	0.090	**−0.673**	**0.047**
**2.0 c/o, 5 Hz**	−0.372	0.324	**−0.677**	**0.045**	−0.266	0.489
**2.0 c/o, 10 Hz**	−0.551	0.124	−0.404	0.281	−0.540	0.134

Changes in music perception scores were defined as the difference between unaided and aided conditions. Positive value indicates the improved scores while negative value indicates the worse scores. Controlling variables were each music perception scores in unaided conditions. The bold indicates significant difference (P < 0.05).

HA benefit for pitch-direction discrimination was irrelevant to psychoacoustic performances (Table 3). The ability to resolve spectral and/or temporal envelope cues did not improve pitch discrimination ability for HA users. However, HA benefit for melody identification was significantly correlated with SMD thresholds (R = 0.835, p = 0.005) and STM detection thresholds (R = −0.848, p = 0.004 for 1.0 c/o and 5 Hz; R = −0.677, p = 0.045 for 2.0 c/o and 5 Hz) (Table 3 and Fig. 4A,B and C). HA benefit for timbre identification was also significantly correlated with STM detection thresholds (R = −0.673, p = 0.047 for 1.0 c/o and 10 Hz) (Table 3, Fig. 4D).

## Discussion

The current study evaluated the relationship between music perception abilities and STM detection thresholds for NH listeners, HA users, and CI users. It has been well established that people with hearing impairment, including CI and HA users, can perceive musical rhythm similar to those with normal hearing^7^. Thus, only pitch discrimination, melody identification, and timbre identification were measured to assess music perception abilities in this study. Our results for their music abilities and psychoacoustic performances were consistent with previously reported data (the K-CAMP subtests: *Jung et al., 2010*, psychoacoustic subtests: *Won et al., 2015*)^14,17^. The present study found that mean STM detection thresholds showed stronger correlations with music perception abilities than SMD or TMD thresholds for all participants (Table 1). However, spectral or temporal modulation cues measured separately showed no correlation with music perception abilities in any group (Table 2). Thus, the hypothesis that a combination of spectral and temporal modulation cues would be more correlated with music perception than spectral or temporal modulation cues separately was supported by the present study.

Next, the relationship between each element of music perception and STM detection performance was examined for each listening group to understand psychoacoustic mechanisms of music perception for hearing impaired listeners. Lower density (0.5 c/o) of STM detection performance contributed to their ability to discriminate pitch-direction for NH listeners, but not for HA or CI users (Table 2). Temporal fine structure might play some roles independently in spectral and temporal processes for pitch perception in hearing impaired listeners because limited spectral and temporal envelope cues were delivered by signal processing of HA or CI system. Drennan *et al*. (2008) have found that 400-Hz Schroeder-phase discrimination test, a test used to measure sensitivity to temporal fine structure, is correlated with pitch discrimination in twenty-four CI users (*r* = 0.52, p = 0.02)^18^. This correlation was independent of spectral ripple discrimination ability (r = 0.52, p = 0.02). Thus, the role of temporal fine structure processing could be important in pitch perception for hearing impairment listeners with HAs or CI.

Generally, melody refers to the overall pattern of frequency changes in a temporal sequence of notes^19^. In this study, melody identification scores were significantly correlated with a combination of spectral and temporal properties of sound for NH listeners and HA users (Table 2). Especially, higher density (2.0 c/o) of STM detection thresholds showed strongest correlation with melody identification scores for NH listeners and HA users. It has been previously suggested that high spectral resolution might be required for melody identification^20^. These results imply that resolution for fast spectral modulation stimuli of STM detection test might have play a primary role in identifying melody. Thus, poor frequency selectivity of CI device might have contributed to the lack of correlation between STM detection performances and melody identification ability (Table 2).

Timbre is often referred to as the color of sound. It has been demonstrated that joint spectro-temporal features are needed for perceptual judgments of timbre^21^. Timbre is encoded via temporal envelope (onset characteristics in particular) and spectral shape of sound. In this study, significant correlations were found between STM detection thresholds and timbre identification scores for all groups (Table 2). Especially, simple linear regression analysis revealed that STM detection thresholds at 1.0 c/o predicted about half of the variance in timbre identification for HA and CI users (Table 2). Previous studies have reported that lower spectral modulation (0.5 c/o) of STM detection tests is significantly correlated with sentence recognition for CI users^14,22^. Thus, spectral resolution performance required to identify timbre might be needed more than identifying speech sound, but less than identifying melody.

Lastly, music perception abilities were compared between unaided and aided conditions for HA users. In addition, correlations between HA benefit for music perception (the difference of music perception between unaided and aided conditions) and aided psychoacoustic performances were evaluated. Since unaided music perception scores generally affect difference in music perception between unaided and aided conditions, partial correlation analysis was used in this study after controlling unaided music perception scores. HA benefit for pitch discrimination was irrelevant to psychoacoustic performances (Table 3), although pitch-direction discrimination score was significantly improved after wearing a HA. Thus, improved pitch-direction discrimination might be due to other factors within the HA device. HA benefit for melody identification was strongly correlated with SMD threshold and STM detection thresholds (Table 3, Fig. 4A,B and C). The significantly improved spectral resolution after wearing a HA might have improved melody identification scores. Benefit for timbre identification was also correlated with STM detection thresholds (Table 3, Fig. 4D). Interestingly, some HA users who had good performance of timbre identification (Fig. 4C) had lower timbre identification scores at aided condition compare to those at unaided condition. Thus, a fitting strategy of HA would be important to preserve spectral and temporal cues for timbre identification.

In the current study, slightly different test paradigms, threshold tracking procedures, and stimulus bandwidths were used for the three different psychoacoustic tests in order to be consistent with our previous works^14,22^. In order to make the modulation dimension of the stimulus be the only factor that varies across three different psychoacoustic tests, one may consider using the exact same bandwidth for the noise carriers, testing paradigm (three-interval, three-alternative forced choice or two-interval, two-alternative forced choice) and the adaptive tracking method for the three different psychoacoustic tests. Secondly, stimuli were presented in the free field for CI subjects, but for NH subjects and HA users, stimuli were presented through an insert earphone. Although all tests were conducted in a double-walled semi-reverberation sound booth, the stimulus presentation in the free field for CI users might have reduced the temporal modulation cues in the high frequency channels due to the potential effect of reverberation^23,24^. However, it should be noted that the bandwidth of the noise carrier for TMD was wideband; thereby, it is unlikely that such reverberation effect in the sound booth might have significantly contributed to TMD thresholds for CI users. Also, it should be noted that the CAMP test was originally developed and validated for music perception for CI users^25^. Nevertheless, a wide range of performance was observed for all three subject groups in the complex-pitch direction discrimination test, melody and timbre recognition tests. Despite these potential limitations, the results of the current study demonstrated that the combination of spectral and temporal modulation cues were more strongly correlated with music perception abilities than spectral or temporal modulation cues measured separately. Also, the current study demonstrated that the STM detection test may be a useful tool to assess music perception performance for hearing impaired listeners fitted with hearing aids or cochlear implants. Further studies with a larger sample size are needed to further understand the psychoacoustic or neural mechanisms involved in music perception performance for these patient populations.

## Methods

### Subjects

**Figure 5:**
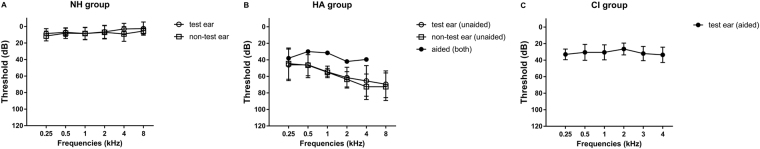
Audiograms for normal hearing (NH) listeners, hearing aid (HA) users, and cochlear implant (CI) users. Panel A shows audiograms for NH listeners. Pure tone thresholds are shown in circle (⚪) for tested ear and in square (□) for non-tested ear. NH listeners had pure tone thresholds better than or equal to 25 dB HL at all frequencies in both ears. Panel B shows audiograms for HA users. Aided pure tone thresholds are shown in black circle (•) for both ears. Unaided pure tone thresholds are shown in circle (⚪) for the tested ear and in square (□) for the non-tested ear. HA users had pure tone average worse than or equal to 40 dB HL for 500 Hz, 1,000 Hz, 2,000 Hz, and 3,000 Hz in both ears. Panel C shows audiograms for CI users. Aided pure tone thresholds are shown in black circle (•) for the tested ear.

A total of 30 subjects (13 males, 17 females) participated in this study, including 10 NH listeners, 10 HA users, and 10 CI users. All subjects were adult native speakers of Korean. Ten NH listeners (4 males, 6 females) with mean age of 27.5 years (range, 20 to 34 years) had pure tone thresholds better than or equal to 25 dB HL at each 500, 1,000, 2,000, 4,000, and 8,000 Hz in both ears. The mean age of the ten HA users (6 males, 4 females) was 47.4 years (range, 21 to 75 years). HA users had more than moderate sensorineural hearing loss (thresholds average for 500, 1,000, 2,000, and 3,000 Hz ≥ 40 dB HL) in both ears. They had at least 12 months of experience with HA prior to participating in the current study. Clinical characteristics of HA users are shown in Supplement 2. The mean age of the ten unilateral CI users (3 males, 7 females) was 50.7 years (range, 23 to 68 years). All CI users were postlingually deafened. They had at least 6 months of experience with CI prior to participating in the current study. Clinical characteristics of CI users are shown in Supplement 3. Pure-tone detection thresholds for these three groups of subjects are shown in Fig. 5. All participants provided written informed consent before completing the study in the Hearing Laboratory at Samsung Medical Center. Approval for this study was obtained from the Institutional Review Board of Samsung Medical Center (IRB No. 2013-06-031). All experiments were performed in accordance with relevant guidelines and regulations.

### Test battery administration

All subjects participated in psychoacoustic and music perception tests. In general, HA and CI users were tested unilaterally with their better ear selected by audiogram in the best-fit listening condition using their own HA or CI. Psychoacoustic tests included STM detection test, spectral modulation detection (SMD) test, and temporal modulation detection (TMD) test. Music perception tests included pitch discrimination, melody identification, and timbre identification. In addition to aided condition, HA users also participated in music perception test at unaided condition. The order of test administration varied within and across subjects. All tests were conducted in a double-walled semi-reverberation sound booth.

### Psychoacoustic tests

A custom made MATLAB® (The Mathworks, Natick) graphical user interface was used to present acoustic stimuli to subjects for psychoacoustic tests. For NH listeners, stimuli were presented monaurally through an insert earphone at an average level of 65 dBA. For HA users, a frequency independent gain equal to half of pure tone average was applied to stimuli. With this gain, stimuli were generally presented at the most-comfortable level (MCL) for HA users. Amplified stimuli were then presented monaurally through an insert earphone. For CI users, stimuli were presented through a loud speaker (HS-50M, Yamaha, Japan) in the sound-field at an average level of 65 dBA. Ear plug was inserted in the non-tested ear during test. CI users sat at 1-m from the loudspeaker. They were asked to face the speaker during the course of the experiment.

#### Spectral modulation detection (SMD) test

SMD was evaluated using a spectral-ripple detection paradigm^26–29^. To create static spectral ripple stimuli (hereafter referred to as static ripple stimuli), 2555 tones were spaced equally on a logarithmic frequency scale with a bandwidth of 354–5656 Hz. Ripple peaks and valley were spaced equally on a logarithmic frequency scale with a ripple density of 1 cycle per octave (c/o). Spectral modulation starting phase for ripple stimuli was randomly selected from a uniform distribution (0 to 2π rad). The stimuli had a total duration of 500 ms.

SMD thresholds were determined using a three-interval, three-alternative forced choice (3-I, 3-AFC) similar to that described in previous studies^14,29^. For each set of three intervals, two intervals contained unmodulated broadband noise and test interval chosen at random with equal a priori probability on each trial contained static-ripple stimulus. An inter-stimulus-interval of 500 ms was used between intervals. Stimuli were equal to the same root-mean-square level. A level rove of ±2 dB (in 1-dB increment) was randomly selected for each interval in the task of three intervals. Three numerically labeled virtual buttons were displayed on the computer screen, corresponding to the three intervals. Subjects were instructed to click on the button corresponding to the interval (i.e., static-ripple stimulus) that sounded different from the other two. For each trial, fresh unmodulated and rippled noise stimuli were used. Each test run began with a peak-to-valley ratio for the rippled stimulus of 20 dB with which most subjects were able to detect the spectral modulation easily. The spectral modulation depth varied adaptively in a two-down and one-up adaptive procedure. After each incorrect response, the spectral modulation depth was increased by a step. It was decreased after two correct consecutive responses. Visual feedback was provided after each trial to indicate the interval that presented the static-ripple stimulus. The initial step size was 2 dB for the first four reversals. The step size was then changed to 0.5 dB for the remaining ten reversals. SMD threshold for each run was defined as the arithmetic mean of the peak-to-valley ratios for the final ten reversal points. The threshold for each subject was calculated as the mean of three testing runs.

#### Temporal modulation detection (TMD) test

TMD test was administered as previously described by Won *et al*. (2011)^30^. The stimulus duration was one second for both modulated and unmodulated signals. For modulated stimuli, sinusoidal amplitude modulation was applied to a wideband noise carrier. For unmodulated stimuli, continuous wide band noise was applied. Modulated and unmodulated signals were gated on and off with 10 ms linear ramps. They were concatenated with no gap between the two signals. TMD threshold was measured using a 2-interval, 2-alternative adaptive forced choice (2I, 2-AFC) paradigm. A modulation frequency of 10 Hz was tested. One interval consisted of modulated noise while the other interval consisted of unmodulated noise. Subject’s task was to identify the interval that contained the modulated noise. A 2-down, 1-up adaptive procedure was used to measure the modulation depth threshold, starting with a modulation depth of 100% followed by decrease in steps of 4 dB from the first to the fourth reversal and decrease of 2 dB for the next 10 reversals. For each testing run, the final 10 reversals were averaged to obtain TMD threshold. TMD thresholds in dB relative to 100% modulation (i.e. 20 ${{\rm{\log }}}_{10}{m}_{i}$) were obtained, where ${{\rm{m}}}_{i}$ indicates the modulation index. The threshold for each subject was calculated as the mean of three testing runs.

#### Spectrotemporal modulation (STM) detection test

The following equation was used based on the previously established technique to create STM stimuli with a bandwidth of four octaves (i.e. 354–5664 Hz)^15^. STM stimuli have been used for assessing psychoacoustic capabilities in recent studies^31,32^.

1 $${\rm{S}}(x,t)={\rm{A}}\times \,\sin \,[2{\rm{\pi }}\times (wt+\Omega x)+\Phi ],$$

In Eq. (1), $x$ is the position on the logarithmic frequency axis in octaves (i.e. *x* = log_2_(*f*/354), here *f* is frequency), and *t* is time on the time axis. Four thousands carrier tones were spaced equally on a logarithmic frequency scale with a bandwidth of 354–5656 Hz. The stimuli had total duration of 1 sec. The spectral envelope of complex tones was modulated as a single sinusoid along the logarithmic frequency axis on a linear amplitude scale. In Eq. (1), ${\rm{A}}$ is the amplitude of the rippled spectral modulation amplitudes, which is defined relative to the flat spectrum. When ${\rm{A}}$ was set to a value between 0 and 1, it corresponded to 0 to 100% spectral modulation of the flat ripple envelope. $\Omega$ is the spectral density in units of cycles per octave (c⁄o). $\Phi$ is the spectral modulation starting phase in radians for carrier tones randomized in radians (range, 0 to 2π). STM stimuli were also modulated in time with modulated spectral envelopes sweeping across the frequency at a constant velocity. In Eq. (1), *w* sets spectral modulation velocity as the number of sweeps per second (Hz), which is referred to as temporal rate in the current study. Positive and negative velocity constructed STM stimuli with spectral modulations (or frequency modulations) that either rose or fell in frequency and repeated over time. As previous study showed no effect of the direction of spectral modulation on STM detection thresholds for normal hearing and hearing impaired listeners^33^, the current study tested a falling direction of spectral modulation alone.

STM test was administered as previously described by Won *et al*. (2015)^14^. To measure STM detection thresholds, a two-interval, two-alternative adaptive forced-choice (2I, 2-AFC) paradigm was used. A silence interval of 500 ms was used between the two intervals. One interval consisted of modulated noise (i.e., test signal) while the other interval consisted of steady noise (i.e., reference signal). Subjects were instructed to choose an interval containing sound like bird-chirping, vibrating, or moving over time and frequency. Subject’s task was to identify the interval which contained a STM stimulus. A 2-down, 1-up adaptive procedure was used to measure STM detection thresholds, starting with a modulation depth of 0 dB followed by decrease in steps of 4 dB from the first to the fourth reversal and decrease of 2 dB for the next 10 reversals. For each testing run, the final 10 reversals were averaged to obtain STM detection threshold. In order to evaluate STM detection performance for different modulation conditions, three different spectral densities (${\rm{\Omega }}$ = 0.5, 1, and 2 c/o) and two different temporal rates ($w$ = 5 and 10 Hz) were tested. Thus, a total of six different sets of STM stimuli were tested. Subjects completed all six different stimulus conditions in a random order. Subjects then repeated a new set of six stimulus conditions with a newly created random order. The sequence of stimulus conditions was randomized within and across subjects. A third adaptive track was obtained if difference between the first two tracks exceeded 3 dB for a given stimulus condition. The final threshold for each STM stimulus condition was the mean of two (or three) adaptive tracks. Before actual testing, example stimuli were played for subjects until they became familiar with the STM stimuli and the task.

### The Korean version of the Clinical Assessment of Music Perception (K-CAMP)

The Korean version of the Clinical Assessment of Music Perception (K-CAMP) test is a test protocol modified from the University of Washington’s Clinical Assessment of Music Perception (UW-CAMP) test to suit Korean^25^. This computer-driven protocol consists of the following three subtests using MATLAB (The Mathworks, Natick) graphical user interface: pitch-direction discrimination test, melody identification test, and timbre identification test. Each test began with a brief training session in which participants could listen to pitch differences and each melody or instrument for familiarity. All stimuli in these music perception tests were presented at 65 dBA for NH listeners and CI users. For HA users, stimuli were presented at MCL using frequency-dependent amplification with a half-gain rule. Complex-tone pitch direction discrimination test used a synthesized piano tone of three different base frequencies (C4 at 262 Hz, E4 at 330 Hz, and G4 at 392 Hz). These tones were synthesized to make envelopes of each harmonic complex. Subjects were asked to select the interval with higher frequency. A one-up and one-down tracking procedure was used to measure the minimum detectable change in semitones that a listener could hear. The step size was one semitone equivalent to a half step on the piano. The presentation level was roved within trials (±4 dB range in 1-dB steps) to minimize level cues. Three tracking histories were run for each frequency. The threshold for each tracking history was the mean of the last 6 of 8 reversals. Threshold for each frequency was the mean of three thresholds from each tracking history.

For melody identification test, 12 melodies familiar to Korean listeners were used. Each melody listed in Supplement 4 had similar features to those used in the UW-CAMP in terms of largest interval, interval width, or number of repeated notes^17^. Melodies retained in the K-CAMP were ‘Airplane’ and ‘Little Star’ corresponding to ‘Mary Little Lamb’ and ‘Twinkle Twinkle’ in UW-CAMP, respectively. They were the same in melodies and rhythms except that they had different titles and lyrics in Korean. Tones were repeated in an eight note pattern at a tempo of 60 beats per minute to eliminate rhythm cues. Rhythm cues were eliminated by repeating long tones in an eight-note pattern. The level of each successive note in the sequence was roved by ±4 dB to reduce loudness cues. Each melody was presented three times. A melody identification score was calculated as percent of melodies correctly identified after 36 melody presentations. Feedback was not provided.

In timbre identification test, sound clips of live recordings for eight musical instruments playing an identical five-note sequence were used. The timbre test was an 8-AFC task. Notes were separated in time and played in the same octave at the same tempo. Recordings were matched for note lengths and adjusted to match levels. Performers were instructed to avoid vibrato. Instruments included piano, guitar, clarinet, saxophone, flute, trumpet, violin, and cello. During actual testing, each instrument sound clip was played three times in random order. Participants were instructed to click on the labeled icon of the instrument corresponding to the timbre presented. Percent of correct answers was calculated after 24 presentations. Feedback was not provided.

### Statistical analysis

Results were analyzed using SPSS 18.0 (SPSS Inc., Chicago, IL, USA). To compare psychoacoustic performance and music perception abilities among the three subject groups, one way analysis of variance (ANOVA) or Kruskal-Wallis test was conducted depending on outcome of normality assumption test. If there was significant differences among the three groups, post-hoc independent t-test or Mann-Whitney test was performed to evaluate differences between two different subject groups (i.e., NH listeners vs. HA users, HA users vs. CI users, and CI users vs. NH listeners) using adjusted p-value of 0.0166 (i.e., 0.05/3) based on Bonferroni correction.

Relationships between psychoacoustic performance and music perception abilities in all 30 subjects were assessed using Pearson’s linear correlation coefficient or Spearman’s rank correlation coefficient. For these analyses, mean STM detection thresholds averaged across six different stimuli conditions were used. Additionally, simple linear regression analysis was used to examine the relationship between music perception abilities and psychoacoustic performance for each subject group. In addition, paired t-test was used to compare music perception abilities for aided and unaided conditions in HA subjects to estimate the effect of amplification on music perception abilities for HA users.

## Electronic supplementary material

supplementary information
