# Author Correction: Relationship between spectrotemporal modulation detection and music perception in normal-hearing, hearing-impaired, and cochlear implant listeners

**DOI:** 10.1038/s41598-020-64150-w

**Published:** 2020-04-29

**Authors:** Ji Eun Choi, Jong Ho Won, Cheol Hee Kim, Yang-Sun Cho, Sung Hwa Hong, Il Joon Moon

**Affiliations:** 1 Department of Otorhinolaryngology-Head and Neck Surgery, Dankook University Hospital, Cheoan, 31116 Republic of Korea; 2 Division of Ophthalmic and Ear, Nose and Throat Devices, Office of Device Evaluation, Center for Devices and Radiological Health, US Food and Drug Administration, Silver Spring, Maryland 20993 USA; 3 Hearing Research Laboratory, Samsung Medical Center, Seoul, 06351 Republic of Korea; 4 Department of Otorhinolaryngology-Head and Neck Surgery, Samsung Medical Center, Sungkyunkwan University School of Medicine, Seoul, 06351 Republic of Korea; 5 Department of Otorhinolaryngology-Head and Neck Surgery, Samsung Changwon Hospital, Sungkyunkwan University School of Medicine, Changwon, 51353 Republic of Korea

Correction to: *Scientific Reports* 10.1038/s41598-017-17350-w, published online 15 January 2018

The original version of this Article contained errors.

Ji Eun Choi and Cheol Hee Kim were incorrectly affiliated with ‘Department of Otorhinolaryngology - Head and Neck Surgery, Samsung Medical Center, Sungkyunkwan University School of Medicine, Seoul, Republic of Korea’.

Affiliations 4 and 5 were omitted from the HTML and PDF versions of the Article.

Additionally, Affiliation 4 was incorrectly given in the Supplementary Information as ‘Department of Otorhinolaryngology - Head and Neck Surgery, Samsung Medical Center, Sungkyunkwan University School of Medicine, Seoul, Republic of Korea’.

Furthermore, Affiliation 5 was incorrectly given in the Supplementary Information as ‘Department of Otorhinolaryngology - Head and Neck Surgery, Samsung Changwon Hospital, Sungkyunkwan University School of Medicine, Seoul, Republic of Korea’

The correct author affiliations are listed below:

Affiliation 1:

Department of Otorhinolaryngology - Head and Neck Surgery, Dankook University Hospital, Cheoan, 31116,Republic of Korea

Ji Eun Choi

Affiliation 2:

Division of Ophthalmic and Ear, Nose and Throat Devices, Office of Device Evaluation, Center for Devices and Radiological Health, US Food and Drug Administration, Silver Spring, Maryland, 20993, United States

Jong Ho Won

Affiliation 3:

Hearing Research Laboratory, Samsung Medical Center, Seoul, 06351, Republic of Korea

Cheol Hee Kim

Affiliation 4:

Department of Otorhinolaryngology-Head and Neck Surgery, Samsung Medical Center, Sungkyunkwan University School of Medicine, Seoul, 06351, Republic of Korea

Yang-Sun Cho & Il Joon Moon

Affiliation 5:

Department of Otorhinolaryngology-Head and Neck Surgery, Samsung Changwon Hospital, Sungkyunkwan University School of Medicine, Changwon, 51353, Republic of Korea

Sung Hwa Hong

Finally, the Abstract contained a typographical error where,

“Ten normal-nearing (NH) listeners, ten hearing aid (HA) users with moderate hearing loss, and ten cochlear Implant (CI) users participated in this study.”

now reads:

“Ten normal-hearing (NH) listeners, ten hearing aid (HA) users with moderate hearing loss, and ten cochlear Implant (CI) users participated in this study.”

These errors have now been corrected in the PDF and HTML versions of the Article.

