# Supplementary material for: Relationship between spectrotemporal modulation detection and music perception in normal-hearing, hearing-impaired, and cochlear implant listeners
Source: Sci Rep. 2018 Jan 15;8:800. doi: 10.1038/s41598-017-17350-w (PMC5768867; doi:10.1038/s41598-017-17350-w)
Supplement: Supplementary file 1 — supplementary information [file 41598_2017_17350_MOESM1_ESM.doc]

**Original Article**

**Relationship between spectrotemporal modulation detection and music perception in normal-hearing, hearing-impaired, and cochlear implant listeners**

Short title: Spectrotemporal Modulation Detection and Music Perception

**Ji Eun Choi, MD^1^, Jong Ho Won, PhD^2^, Cheol Hee Kim^3^, Yang-Sun Cho MD, PhD^4^, Sung Hwa Hong, MD, PhD^5^, Il Joon Moon, MD, PhD^4^**

^1^Department of Otorhinolaryngology - Head and Neck Surgery, Dankook University Hospital, Cheonan, Republic of Korea, ^2^Division of Ophthalmic and Ear, Nose and Throat Devices, Office of Device Evaluation, Center for Devices and Radiological Health, US Food and Drug Administration, Silver Spring, Maryland, 20993, ^3^Hearing Research Laboratory, Samsung Medical Center, Seoul, Republic of Korea, ^4^Department of Otorhinolaryngology - Head and Neck Surgery, Samsung Medical Center, Sungkyunkwan University School of Medicine, Seoul, Republic of Korea, ^5^Department of Otorhinolaryngology - Head and Neck Surgery, Samsung Changwon Hospital, Sungkyunkwan University School of Medicine, Seoul, Republic of Korea

**Financial Disclosures/Conflicts of Interest:**

The authors have no conflicts of interest to declare.

Correspondence: Il Joon Moon, MD, PhD

Department of Otorhinolaryngology-Head and Neck Surgery, Samsung Medical Center, Sungkyunkwan University School of Medicine, 81 Irwon-ro, Gangnam-gu, Seoul 06351, Republic of Korea. Tel: +82 2 3410 3579. Fax: +82 2 3410 3879. E-mail: moonij@skku.edu

**Supplement 1. Music perception abilities of HA users at unaided and aided conditions.**


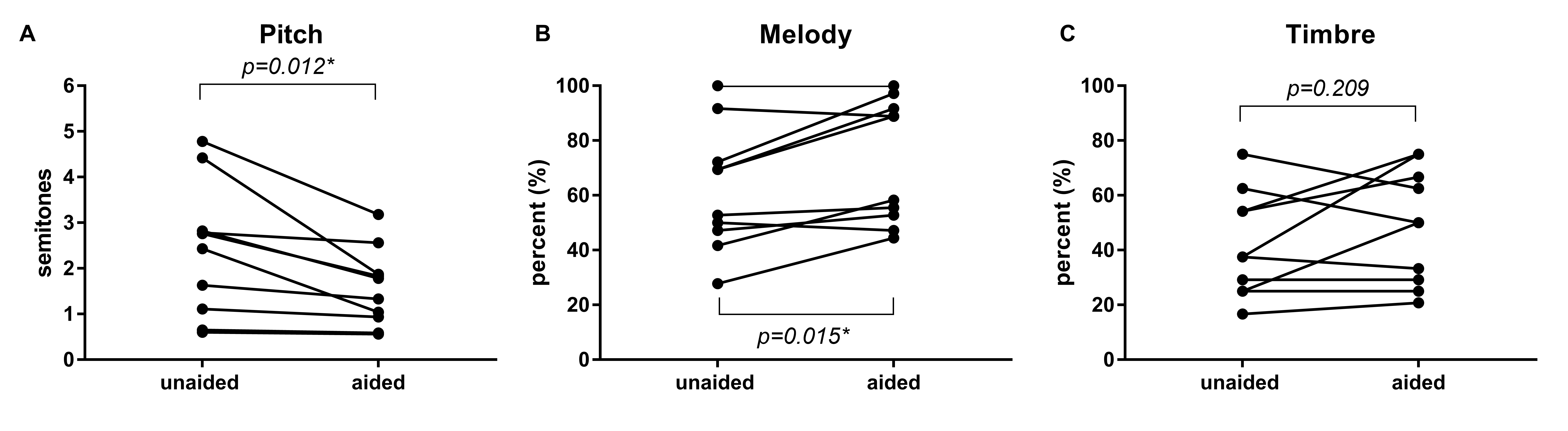


Results of music perception abilities at unaided and aided conditions are shown for pitch (A), melody (B), and timbre (C). Asterisk (*) indicates significant difference between two conditions in paired t-test analysis (P < 0.05).

**Supplement 2.** Clinical information of Hearing Aid users.

| **HA** | **Duration of**  **HA use (months)** | **Manufacturer** | **Model** | **Type** |
| --- | --- | --- | --- | --- |
| HA 1 | 60 | GN Resound | essence 10P | CIC |
| HA 2 | 49 | Siemens | Motion 501 | CIC |
| HA 3 | 12 | Siemens | INTUIS | CIC |
| HA 4 | 58 | Siemens | Cielo 2 | CIC |
| HA 5 | 129 | GN Resound | DOT 10 | RIC |
| HA 6 | 28 | Starkey | Destiny 400 | CIC |
| HA 7 | 51 | GN Resound | essence 10P | CIC |
| HA 8 | 80 | Phonak | Certerna art P | CIC |
| HA 9 | 32 | GN Resound | DOT 2 | RIC |
| HA 10 | 14 | Oticon | ria Pro mini | BTE |

**Supplement 3.** Clinical information of cochlear implant subjects.

| **CI** | **Duration of implant use (months)** | **Sound processor** | **Implant type** | **Number of channels** | **Speech processing strategy** |
| --- | --- | --- | --- | --- | --- |
| CI 1 | 59 | Harmony | HiRes 90K HiFocus | 13 | HiRes /S |
| CI 2 | 94 | Auria | HiRes 90K HiFocus | 16 | HiRes /S |
| CI 3 | 86 | freedom | CI24RE(CA) | 22 | ACE |
| CI 4 | 33 | OPUS2 | Flex(soft) | 12 | FS4 |
| CI 5 | 29 | N5(CP810) | Freedom(contour advance) | 22 | ACE |
| CI 6 | 24 | OPUS2 | Flex(soft) | 12 | FS4 |
| CI 7 | 20 | CP810 | CI422 | 22 | ACE |
| CI 8 | 124 | Auria | HiRes 90K HiFocus | 16 | Hires /P |
| CI 9 | 13 | Rondo | Flex(soft) | 12 | FS4 |
| CI 10 | 62 | N5(CP810) | N5(CI512) | 22 | ACE |

**Supplement 4.** The 12 familiar melodies selected for the test and their frequency ranges.

| **Melody** | **Range (Hz)** | **Largest interval^*^** | **Interval width^**^** | **Longest repeated note** |
| --- | --- | --- | --- | --- |
| Little star (Twinkle Twinkle) | 262–440 (C4–A4) | 5th | 9 | 2 |
| School bell | 262–440 (C4–A4) | 3rd | 9 | 4 |
| Pongdang | 262–440 (C4–A4) | 5th | 9 | 3 |
| Butterﬂy | 262–392 (C4–G4) | 3rd | 7 | 4 |
| Airplane (Mary Little Lamb) | 262–330 (C4–G4) | m3rd | 7 | 4 |
| Bicycle | 330–440 (E4–A4) | m3rd | 5 | 8 |
| Arrirang | 294–494 (D4–B4) | 3rd | 9 | 3 |
| Cow | 220–494 (A3–B4) | 4th | 14 | 4 |
| Rabbit | 262–523 (C4–C5) | 4th | 12 | 2 |
| Moon | 262–523 (C4–C5) | 5th | 12 | 4 |
| Spring | 330–660 (E4–E5) | 4th | 12 | 4 |
| Gaenari | 262–523 (C4–C5) | 4th | 12 | 2 |

*^*^Largest interval in the melody; m, minor.*

*^**^Interval width: range (in semitones) between the highest and the lowest notes in the melody*
